# Role of fluid cohesiveness in safe swallowing

**DOI:** 10.1038/s41538-019-0038-8

**Published:** 2019-04-03

**Authors:** Katsuyoshi Nishinari, Mihaela Turcanu, Makoto Nakauma, Yapeng Fang

**Affiliations:** 1 Glyn O. Phillips Hydrocolloids Research Centre, School of Food and Biological Engineering, Hubei University of Technology, Wuhan, 430068 China; 2 Product & Process Engineering Center- Germany, Pharmaceuticals Division, Fresenius Kabi Deutschland GmbH, Daimlerstrasse 22, 61352 Bad Homburg, Germany; 3 San-Ei Gen F.F.I., Inc., 1-1-11, Sanwa-cho, Toyonaka, Osaka 561-8588 Japan; 4 Department of Food Science and Engineering, School of Agriculture and Biology, Shanghai Jiao Tong University, Shanghai, 200240 China

**Keywords:** Risk factors, Quality of life, Biophysics

## Abstract

In patients with dysphagia, it has been a practice to thicken fluid food to prevent aspiration—the transport of a bolus into the trachea instead of the oesophagus. In these patients, aspiration is a risk behaviour and is closely related to pneumonia (caused by the aspiration of oral bacteria into the lungs). Since excessive thickening of fluids can cause adverse effects, such as lowering the palatability of food, subsequent reduction of liquid intake, dehydration and malnutrition, identifying the optimum thickening level is vital. Thickening might not only increase fluid viscosity, but could also modify its cohesiveness, which is another key factor affecting aspiration. Even though cohesiveness is more of a concept than a well-defined measurable parameter, this property describes the degree of coherency provided by the internal structure of a material against its fractional breakup. In fluids, this concept is less explored than in solids, powders and granules, and during the last decade few scientists have tackled this topic. Although the role of cohesiveness in the swallowing of heterogeneous solid foods is briefly overviewed, the aim of the present paper is to introduce the concept of cohesiveness for a relatively homogeneous fluid bolus and its effect on swallowing. Cohesiveness is highly correlated with the extensibility and yield stress of the fluid, suggesting that a high cohesiveness could have an important role in preventing aspiration.

## Introduction

Given that the average life expectancy is becoming longer, the long life in a good health is required to reduce the probability of lying in bed all day long. With the advent of aged society, the number of persons having difficulties in mastication and deglutition is increasing, and pneumonia caused by aspiration of food through the airway,^1–5^ is now the third most important cause of death in Japan after malignant neoplasm and heart disease in elderly (>65 years old).^6^

Let’s look back in the history. All the solid foods are masticated to be broken down into small fragments and mixed with saliva until optimal lubrication before being swallowed^7^. This mouth process model has been taken into account by many texture research groups as a starting point to understand the oral processing of food.^8–10^ In a well-known comedy of Molière “Le Bourgeois Gentilhomme (The Middle Class Gentleman)”, a rich snob training on how to pretend to be an authentic noble is taught how to pronounce the sound “f” by an employed philosophy master, as *“*The F, by pressing the upper teeth against the lower lip: F”. This kind of teaching was thought to be funny because this was obvious and not necessary to be taught to an adult person. In the age of Molière, the audience was pleased to see this scene to mock a parvenu. However, in our era, this is not funny at all and a developed discipline of linguistics and speech therapy helps people with the difficulties in the pronunciation and swallowing, which are closely related to dysphagia.^11–13^

Hutchings–Lilllford swallowing model was proposed in 1988, but not so many food professionals could recognize directly its importance, since it was only outlining the food oral processing, which healthy individuals do unconsciously. This was as if the audience of Molière’s comedy would not recognize the significance of the pronunciation method in the 17th century. Fortunately, the editor Bourne of the Journal of Texture Studies was a man of foresight and published it. Even though it took a long time before its importance was recognized, the Hutchings–Lilllford model was used by many researchers as a starting point for discussion in the first Food Summit “Food texture: perception and measurement” in 1999,^14^ which was published in Food Quality and Preference). Although the model of Hutchings and Lillford is very versatile and schematizes concisely the dynamic nature of the oral processing, it still remains a simplified model, since it does not quantitatively specify the force, the distance or the times involved in the process and does not take into account the effect of smell and taste on the mastication behaviour or salivation. The first International Congress on Mastication and Health was held jointly with the 13th Meeting of the Japanese Society for Mastication Science and Health Promotion in Yokohama in September 2002.^15^ The special issue of *Journal of Texture Studies* (Vol. 35(4), 2004) was published collecting six papers read at this conference. After the successful review of food oral processing (FOP) by Chen^8^ and an international conference (5–7 July 2010, Leeds, UK), a subsequent international conference is organized every 2 years, activating more interaction among specialists of different disciplines: medicine, physiology, dentistry, speech therapy, leading to more active research in the field of sensations, texture, taste and odour, all with the same aim in understanding better the mastication and swallowing patterns.^16–18^

It has empirically been acknowledged that the risk of aspiration during swallowing can be reduced by thickening liquids such as water, tea or fruit juice with different starch and gum-based thickeners.^3,19–21^

In USA, the National Dysphagia Diet (NDD) recommends the use of shear viscosity at a particular shear rate of 50/s and at 25 °C in order to classify the fluid thickness level as: “thin” (1–50 cP, “nectar-like” (51–350 cP), “honey-like” (351–1750 cP) and “pudding-like” (>1751 cP), since it is assumed that this classification could be easily understood in hospitals and healthcare organizations.^19^ Note that the viscosity unit of 1 cP is equivalent to 1 mPas in the International System of Units. However, since in other countries, especially in Asian countries, these terms are not so clearly understood, a more recent attempt, the International Dysphagia Diet Standardization Initiative (IDDSI), founded in 2013, introduced the terms “thin” (level 0, flows like water), “slightly thick” (level 1, thicker than water requires a little more effort to drink than thin liquids), “mildly thick” (level 2, flows off a spoon), “moderately thick” (level 3, will not hold shape its shape on a spoon), and “extremely thick” (level 4, holds shape on spoon) to represent the thickness of drinks.^23^

It should be noted that the term “thick” is usually used in sensorial evaluation mainly induced by the attribute of the fluid viscosity, while the “viscosity” is rigorously defined in fluid mechanics both for Newtonian and non-Newtonian fluids. In psychophysics, the relation between sensorily evaluated thickness and instrumentally measured viscosity has been extensively studied, and these two terms are not the same, although high correlation is found in well-designed experiments.

Even though there is currently a lot of discussion on choosing the right thickness terminology, when considering the safe swallowing, there seems to be a common consensus on the role of increasing viscosity. But to which extent the viscosity should be increased? Even healthy persons do not like to sip thickened fluids if they are too thick.

It is known that patients with dysphagia consume less-thickened liquids than if they were to consume them un-thickened. It is due to the combinatorial effect of worsened flavour release and worsened thirst quenching ability caused by thickening agents.^19^ While it is widely recognized that a thickening liquid reduces the risk of aspiration, it is also inducing the satiety feeling, which, in healthy individuals could be an effective strategy to prevent obesity, but for dysphagic patients, who need more hydration and more nutrients, could be unfavourable.^5^

Apart from viscosity, the integrity of the bolus during swallowing plays, as well, a role in reducing the risk of aspiration. In other words, it is not only necessary that the food bolus travels slower through the pharynx, providing more time to the epiglottis to secure the airway, but it is necessary that it preserves its consistency and does not adhere to the pharynx walls or split into smaller particles.^5,24–26^

Therefore, in the present paper, the significance of the cohesiveness, in addition to the viscosity of fluids, in the dysphagia management is discussed.

## What is cohesiveness?

It might be, firstly, necessary to draw attention to the misconception of cohesiveness in the food science literature. Bourne^27^ was impressed by how many texture terms (more than 450, see ref. ^28^) are used in Japan. However, as mentioned in his textbook, “cohesiveness” is not included in the most frequently used ten texture terms, neither in USA, nor in Japan or Austria, although conceptually, it is thought to be an important term to describe food texture. A possible reason could be that the significance of the term is difficult to be grasped or represented.

In order to define cohesiveness of a fluid bolus, an overview of the historical usage of this term for solid particles (such as powders or granules) is necessary. The term “cohesiveness” was first defined in food science as the strength of the internal bonds making up the body of the product^29^ and it was determined experimentally as the ratio of the area under the curve of the second bite to that of the first bite in texture profile analysis (TPA).^30^ However, the physical meaning of this concept was not clear, because the *x*-axis in TPA was the time, and Peleg^31^ changed it to the deformation, so that the area under the curve would represent the work or energy.^10,32^ Back in 1977, Peleg used the term “cohesiveness” to represent the state in which particles stick to each other and agglomerate to form a lumped mass.^26,33,34^ Therefore, an increase in cohesiveness could induce a decrease in flow-ability.

Flow-ability of solid particles is characterized quantitatively by the flow index, the ratio *ff*_c_ of unconfined yield strength to the consolidation stress. The larger value of *ff*_c_ indicates the higher flow-ability, where the consolidation stress and the unconfined yield strength are determined in the uniaxial compression test of solid particles filled in a hollow cylinder and those without a hollow cylinder.^35^ The flow behaviour of solid particles is classified by the value of the flow index *ff*_c_, as: *ff*_c_ *<* 1 not flowing, 1 < *ff*_c _*<* 2 very cohesive, 2 < *ff*_c_ *<* 4 cohesive, 4 < *ff*_c_ *<* 10 easy-flowing, 10 < *ff*_c_ free-flowing.^35^

The flow-ability of solid particles has been evaluated by modified Jenike’s shear cell, which is used for the analysis of flow of the powders and granules in bins and hoppers.^36^ It was found that the flowing behaviour of solid particles depends on particle size and distribution, particle shape, particle surface roughness and friction, surface electric charges, voidage, moisture and temperature.^33^

Fluid cohesiveness may be quantified also by the degree of fragmentation of a fluid which flows out from the space such as a tube or a nozzle where it has been confined, as is commonly done in spray drying process. Although cohesiveness of powder has been studied extensively in spray drying technology using a simple index called Hausner ratio defined as the ratio of the bulk density and tap density,^37,38^ the cohesiveness of a fluid droplet before drying into a powder state has not been experimentally determined as far as the authors are aware.

Prinz and Lucas^25^ pointed out the importance of cohesiveness in the oral processing. They proposed an optimization model: food is crushed into small particles, by mastication, and bound together by salivation when viscous cohesion is promoted, leading to bolus formation. By assuming that the particles are spherical, the cohesive force, *F*_V_ − *F*_A_, was defined as the difference between the viscous force and the adhesive force. The term cohesive force represents the force that makes cohere or stick the like particles each other while adhesive force signifies the force which makes particles cohere or stick to the organs of oral cavity. Prinz and Lucas assume the viscous force as an attractive force between two disc-like surfaces, the gap of which is filled with saliva, where the disc-like surfaces are on the imagined section in the centre of this spherical particle. If swallowing is delayed, particles are separated by excessive saliva and the cohesion is reduced. Prinz and Lucas^25^ examined the validity of the model for humans using Brazil nuts and raw carrots. They found that both foods, in spite of the difference in mechanical characteristics, are swallowed at approximately the same number of chews, when the cohesive force becomes maximum. According to their model, the time at which the cohesive force becomes maximum is the optimum moment for swallowing. Although the concept of viscous force and adhesive force introduced by Prinz and Lucas^25^ is interesting and may be a starting point for the further clarification, it is still difficult to understand and to apply these concepts for real boli at molecular level.

Examining the particle size distribution of the bolus produced from 3 g petal wheat-flake cereals collected at different stages of mastication sequences, Peyron et al. (2011)^39^ found that the cohesiveness increased from the middle stage to the final stage just before swallowing while the particle size and the hardness of the bolus decreased with progressive mastication. They also found that the dryness sensation increased just like the cohesiveness, which was attributed to the absorption of saliva into the bolus. This behaviour might be observed for boli obtained with wheat-flake cereals, but may not be generalized to other solid foods containing more water. Peyron^39^ recognized a narrow variability in particle size of the food bolus just before swallowing, in contrast to a broader variability of the physiological parameters.^40–42^ However, since Fontijn-Tekamp et al. (2000)^43^ reported a greater variability of the particle sizes in the bolus, Peyron et al.^39^ stated that other determinants (e.g. rheological/saliva content) are probably involved in the swallowing threshold concept. Jalabert-Malbos et al.^40^ found that the mean particle size of food bolus should be smaller than a certain critical value, which supports Hutchings and Lillford’s model, suggesting that this critical size should be small for hard brittle foods (e.g. peanuts) and much larger for softer foods. The reason for the larger critical size for softer foods was attributed to the lower liability of softer foods to injure the upper digestive mucosae, which is consistent with findings for grittiness sensation. Harder particles are felt gritty at a smaller size than softer particles.^44–46^

However, it is still difficult to identify only one critical factor triggering the swallowing considering that swallowing threshold is probably an integrative process combining the perception of various bolus properties^39^.

Chen and Lolivret^24^ questioned the statement of swallowing at the maximum cohesive force introduced by Prinz and Lucas^25^ because swallowing muscles will have to work much harder to create a high enough oral pressure to push bolus through the oropharyngeal system and, therefore, it is not the logical choice. They have found that the oral residence time for 28 different fluid foods increased with increasing apparent shear viscosity which is in good agreement of Taniguchi et al.^47^ who observed that the total swallowing time and oral ejection time increased in the order of liquid < syrup < thin paste < thick paste. The swallowing evaluation was shown to be correlated well with the apparent shear viscosity, but even higher correlation was found between swallowing and stretch-ability. Chen and Lolivret^24^ concluded that the bolus extensional behaviour could be the key determining factor in triggering the swallowing and that the incorporation of a sufficient amount of saliva improves the flow-ability and stretch-ability of the bolus for a safe and comfortable swallowing. This was recently supported by Morell et al.^48^ This assumption clearly denies the statement of Prinz and Lucas^25^ in which excessive saliva will flood the bolus and may induce the risk of aspiration. It should be noted that the swallowing easiness was evaluated by Chen and Lolivret^24^ in healthy young subjects, who assessed water as the easiest fluid to be swallowed. This appears contradictory to many papers reporting the effectiveness of thickening agents in preventing aspiration.

Devezeaux de Lavergne, van Delft et al.^49^ supported the hypothesis of Chen and Lorivret^24^ that flow-ability of bolus is more important than high cohesiveness, since they observed that Young et al.,^50–52^ experiments on biscuits failed in confirming the hypothesis that cohesiveness is a deciding factor for swallowing, although Young et al. found the validity of this hypothesis for cereal flakes. Based on their observations of dynamic change of boli prepared from emulsion-filled semi-solid food model, gels were perceived either as creamy or grainy in the last stage of oral processing. Only gels perceived as creamy revealed a high bolus flow-ability. Devezeaux de Lavergne, Derks et al.^53^ compared the oral processing of sausages and found that both, long-duration and short-duration eaters, perceived the same sausage similarly in the early stages of oral processing, but started to perceive its texture differently from the middle of oral processing towards the end. They found no compensation for a shorter eating time of the short duration eaters by applying a higher chewing frequency or an increased muscle effort. They found that the incorporation of saliva into bolus was more significant for longer duration eaters which is in agreement with Tarrega et al.^54^ They also found that although roundness of fragments was similar at the moment of swallowing in both groups, short-duration eaters generate less broken-down hard sausage boli containing fewer and larger fragments at all eating times than long-duration eaters. Thus, between the two groups, the bolus properties were found different at the end of mastication.

As already mentioned, food cohesiveness was first determined experimentally in the texture profile analysis (TPA).^30^ Since then, some papers have been published and erroneous applications of TPA to fluids have been practiced, unfortunately. The cohesiveness, defined as the ratio of the energy required for the second compression to that of the first compression in TPA, has some meaning only for solid foods.^10,32,55^ When a rubber-like material is masticated, the cohesiveness is found very close to one,^56^ indicating that such food model recovers the initial shape and size after biting. Although such a too rubbery-like food is not disintegrated at all and it may not be liked, the significance of the cohesiveness was clearly shown in this experiment. The ideal elasticity in mechanics has two aspects: (1) the deformation is instantaneous (no time delay), (2) total recovery of the original shape and size after removing the force. Since plastic solids do not recover the initial size and shape, the cohesiveness determined by TPA procedure is zero because the area in the second bite is zero if the solid is purely plastic and not elastic. In solid particles, such as powders and granules, cohesiveness is caused by agglomeration of particles and higher cohesiveness indicates the poor flow-ability as mentioned above.

Rosenthal^57^ showed that the cohesiveness of a starch gel model decreased from about one to one-third with increasing compression from 25 to 90%, and he interpreted this reasonably consistent with the original significance of the cohesiveness proposed by the originators of TPA: “a direct function of the work needed to overcome the internal bonds of the material”.^58^ But, he warned about the misappropriation of TPA data since TPA is an easy experiment and the number of papers is rising. He recommends performing TPA at higher compression speed than 2 mm/s where he observed leveling off of the hardness for his model starch gels but below that speed the hardness obtained is much smaller. In most cases TPA is performed to find the correlation with the sensory evaluation, therefore TPA parameters obtained at lower compression speed should not be used.^10^

Young et al.^50^ tried to find the swallowing trigger for biscuits with three different sugar/fat ratios, B1 the lowest sugar and B3 the highest sugar content. They preferred to use back extrusion (BE) method proposed by Osorio and Steffe.^59^ They observed that the consistency index, *η*, by BE and the peak force by TPA decreased significantly, while the cohesiveness by TPA increased significantly from the middle stage to final stage of processing. They also found that flow behaviour index, *n*, determined by BE increased slightly from the middle stage to final stage of processing. Since both, the cohesiveness and the consistency index of the boli at the swallowing point decreased with increasing sugar content, they concluded that “cohesiveness” is not a swallowing trigger for the biscuits as it was significantly different for the three biscuit recipes, thus questioned the optimum swallow model of Prinz and Lucas.^25^ Since the study mentioned above was done based on only one single subject (a 25-year- old healthy woman),^51^ examined the same problem employing five subjects using BE and modified TPA for a biscuit with the same sugar/fat ratio as B1. They determined the bolus yield stress by BE at each stage, and found that the yield stress decreased from early stage to the point of swallow. This yield stress was found to be comparable to the static yield stresses of cereal bolus at the swallowing point (1.3–4.3 kPa), which was determined by vane geometry.^60^ Authors found many inter-individual differences in perceived texture and breakdown paths, mastication period, number of chews, and chewing frequency, bolus mass, yield stress, peak force, “adhesiveness”, “cohesiveness”, and description of bolus properties. The authors concluded that one single parameter determining the point of swallow could not be found for their biscuit recipe. Young^52^ raised the advantage of BE because it could test boluses which still contain larger particles and be carried out using the same simple testing apparatus as TPA. The present authors would not like to discourage him and rather wish the further development of the method, but the consistency index and the flow index that they obtained may have meaning only when the bolus consists of smaller particles. Depending on the mastication ability, some boli contain larger particles and in such cases the diameter of the cylinder and the gap should be enlarged, and then these parameters may not be obtained. This is similar to the usage of falling sphere or needle viscometer where the diameter of sphere or the needle should be increased to get an average rheological value for inhomogeneous bolus containing larger particles.^3^

If this TPA method is used for fluids it leads to a contradictory conclusion: the “cohesiveness of water” is exactly one (water conforms to the shape of the container, as has been known from ancient times), and much larger than that for xanthan solutions, while the “cohesiveness of xanthan solution” decreases with increasing concentration of xanthan. This is obviously illogical.^61^ It can be predicted without measurement, but since such a strange situation prevailed in Japan, these authors were obliged to write such a paper.

Even though starch-based thickening agents are still widely used in dysphagia products, in the last years gum-based thickeners gained more and more attention, namely due to their ability to form thicker fluids at considerably lower concentration than starches and to provide higher thickness with lower oropharyngeal residues.^62^ Another benefit of gums in dysphagia management is the lower degradation by the salivary enzymes during the oral processing, which makes them safer to swallow. Therefore, in the present paper, three polysaccharide thickening agents, xanthan, locust bean gum and guar gum (GG) were selected as model boli and the significance of cohesiveness is discussed based on their fluid extension, acoustic and video-fluorographic analysis of swallowing. The decision to use gums instead of starches for such illustration is that starch is as well, prone to show thixotropic behaviour and viscosity changes during storage, while xanthan gum and galactomannan gums are free from retrogradation.

The main focus of this work is not only to discuss the role of cohesiveness as a triggering factor of swallowing, but also its effect on the swallowing speed of the bolus through the pharynx and therefore, its ability in preventing aspiration. Consequently, this study can be considered as a basis to understand the role of cohesiveness using an ideal homogenous food bolus model which does not require chewing and it is not strongly affected by the salivary enzymes, since chewing and enzymatic degradation would complicate the picture of the swallowing mechanism, as already discussed above. To develop further understanding, it is surely necessary to study more in depth non-homogeneous boli since they represent a vast majority of the real fluids.

## Extensional properties and yield stress of gum-based fluid models

For many years, rheology showed its relevance in food bolus characterization, as it was directly linked to the performance of the swallowing process. Food bolus flow is certainly a dynamic process, strongly dependent on the fundamental composition of food and on the ability to change its rheological behaviour during the formation of the bolus in response to the applied forces during oropharyngeal processing. During swallowing a food bolus is subjected to both, shear and extensional flows.

However, the focus is usually centred on the measurement of shear viscosity, despite the fact that the elongational properties of food boli might provide important insights on the dynamics of food during the swallowing process. Even though in food industry the elongational properties are still not extensively studied and bolus elasticity is rather ignored in the current guidelines, it is assumed that higher extensional properties of food bolus could be correlated to higher cohesiveness during the oral processing. This is particularly important since a cohesive bolus will fracture/breakup less during the pharyngeal phase of the swallowing, which could decrease the risk of aspiration for patients with dysphagia.^4,63^

Extensional deformation is well studied theoretically, but is less implemented experimentally to fluids due to the technical limitations in what concerns the availability of experimental instruments. Capillary breakup extensional rheometer (CaBER) is commercially available (ThermoHaake Gmbh, Karlsruhe, Germany) for the extensional characterization of fluids. The technique uses the evolution of a filament diameter over time in order to extract extensional (elastic) properties of fluids.

Turcanu^64^ studied the extensional properties of xanthan gum and of other polysaccharide solutions which are widely used in dysphagia products. Xanthan gum (XG), for instance, is a microbial polysaccharide having a cellulose backbone and five saccharide side chains, widely used for rheological control because of its high viscosity even at very low concentrations and a relative insensitivity to the change in temperature and ionic strength in comparison with other thickening agents.^65,66^ Enormous number of papers have been published on the XG conformation in various solutions, which still constitutes a hot research topic.^67–69^ GG and Locust bean gum (LBG) are plant polysaccharides with mannose backbone having one galactose side group per two mannose residues in GG and one galactose side group per four mannose residues in LBG, widely used as thickening agent in food industry.^70^

Commercial gum powders were added in appropriate amount to deionized water in order to produce 100 mL solutions of 0.5 wt%. The powder was added to water at 50 °C while mixing on a magnetic stirrer. The dispersion was stirred mildly for 15 h at room temperature (25 ± 2 °C), stored at 4 °C at least for 1 day before the measurement and kept at room temperature for 1 h before measurement.

Each fluid sample was injected between the two parallel plates of the capillary breakup extensional rheometer with the diameter, *D*_0_ = 6 mm, separated by an initial gap, *L*_0_ = 3 mm. The bottom plate was fixed, while the upper plate moves up at 0.16 mm/s within 50 ms, until it reaches the final length *L*_f_ = 11 mm. An attached high-speed camera (Photron Fastcam Mini UX100, Phototron Ltd, UK) was used to record the filament evolution during capillary thinning.

**Fig. 1:**
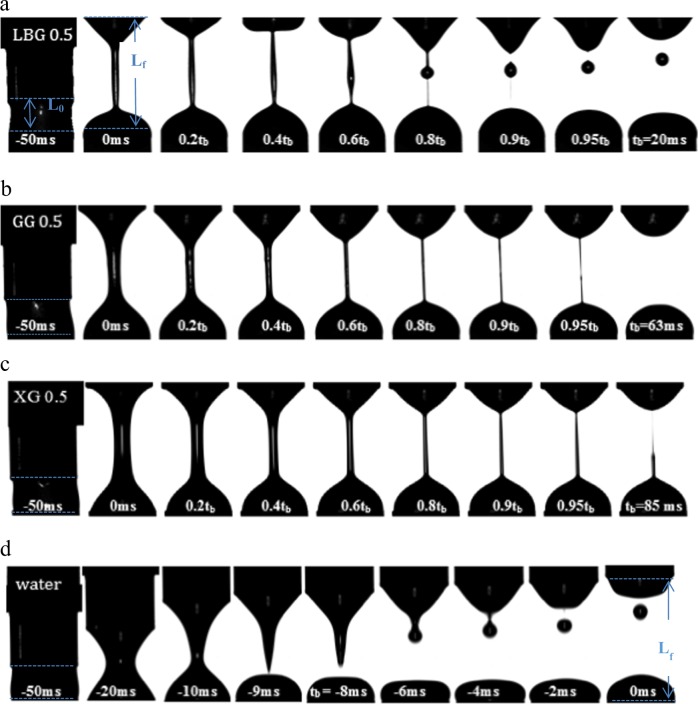
**a** Extensional deformation of 0.5% locust bean gum solution. **b** Extensional deformation of 0.5% guar gum solution. **c** Extensional deformation of 0.5% xanthan solution. All the measurements were performed at 25 °C. Solutions are extended from the initial length *L*_0_ (=3 mm) with the initial diameter *D*_0_ (=6 mm) to the final length *L*_f_ (=11 mm) at 0.16 mm/ms. Each set of images presents the filament at rest (−50 ms), at the initial moment of the extensional experiment (0 ms), and at 6 time frames preceding the filament rupture time, *t*_b_ (breakup time). **d** Extensional deformation of water (Turcanu^64^ and unpublished)

Figure 1 illustrates the filament evolution of XG, GG and LBG solutions together with water during uniaxial elongation. As can be observed, the breakup time of LBG solution is much shorter than the ones of XG and GG solutions and the filament diameter becomes thinner at shorter time. It is also interesting to notice that a 0.5% LBG solution forms a satellite droplet during breakup, while a 0.5% XG and GG solutions creates a filament that preserves its integrity until the end of the capillary process. The formation of a satellite droplet during breakup was also observed for xanthan solution with lower concentrations.^64^

These examples are shown since the spinnability (thread forming ability) has attracted much attention from the part of Japanese rheologists since there are many food fluids, such as okra and mucilaginous fluid covering *natto* (fermented soybeans) that show this behaviour. When a glass rod is immersed into a liquid and then pulled up vertically, even sugar syrup which is very close to a Newtonian fluid shows a thread because a liquid adhering on the glass rod is flowing down, and when all the liquid flows down, the thread breaks. However, when the fluid has a certain elasticity, the fluid extends by rubber elasticity and when the fluid breaks the suspending fluid climbs up because of the elastic recovery. Therefore, the maximum extendable length of the fluid depends on the ratio of the viscosity and elasticity, which is known as the relaxation time.^71^

Extracting the relaxation time during uniaxial elongation of dysphagia-designed fluids in CaBER experiments seems promising, but is limited by the formation of highly non-cylindrical and poorly reproducible filaments during uniaxial elongation.

More recently, Turcanu et al.^72^ proposed the axial force measurement during uniaxial elongation as an alternative for monitoring the elongational behaviour of dysphagia-oriented thickened fluids, both before and after the contact with human saliva. Fluids prepared with commercially available powder thickeners were tested, both pure and mixed with reconstituted salivary α-amylase fluid at a ratio of 10:1. Elongational flow behaviour and the full filament profile were monitored and the axial forces during the stretching were recorded.

Alpha-amylase solution was prepared by adding alpha-amylase lyophilized powder to deionized water. Powder activity, as guaranteed by the supplier, was 280 U/mg of protein at 37 °C. The necessary amount of alpha-amylase powder was diluted in water in order to obtain the desired activity of 125 U/ml, previously reported as average activity of human saliva in healthy individuals.^73^

**Fig. 2:**
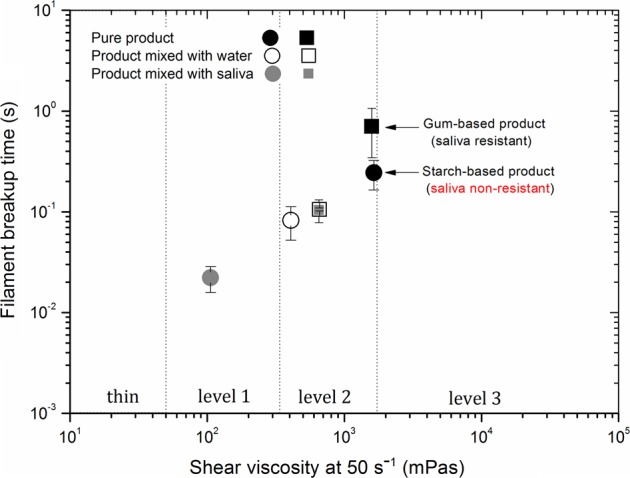
Effect of 10:1 water or α-amylase solution addition on the capillary breakup time and shear viscosity at 50/s of a starch-based and a gum-based dysphagia product (replotted from ref. ^64^). The viscosity range is classified into four stages: Thin, Level 1, Level 2 and Level 3 according to the National Dysphagia Diet (NDD)

As shown in Fig. 2, the filament breakup time increases with increasing the fluid consistency indexed by the shear viscosity observed at 50/s. The thinnest level 1 called nectar-like fluid shows the shortest filament breakup time, while the thickest level 3 called pudding-like fluid shows the longest filament breakup time.^64^ In addition, Fig. 2 shows the effect of addition of alpha-amylase solution on both, shear and extensional properties of a starch-based and a gum-based thickening products. While the filament breakup time and shear viscosity of the starch-based thickener decreased drastically in the presence of saliva, for the gum-based thickener the alpha-amylase addition had only a slight dilution effect, very similar with water effect on both breakup time and shear viscosity.^63,64^ The drastic decrease in viscosity of the starch-based product in the presence of alpha-amylase was expected, since starches are known to be fast hydrolysed by alpha-amylase while XG is not affected. Additionally, due to the lack of elasticity of the starch product, the alpha-amylase addition also affected the fluid behaviour during elongation, by decreasing the filament break-up time and, eventually leading to the creation of satellite droplets, as observed for LBG solution and water in Fig. 1a, d, respectively. More details regarding the testing methodology can be found in Turcanu et al.^72,74^

Turcanu et al.^74^ also found that the effect of real human saliva and the effect of artificial saliva on the rheology of a starch-based fluid are similar in shear, while human saliva has a much pronounced elasticity in elongation. This difference was attributed to the presence of mucin, a glycoprotein contained in human saliva. Since saliva is known to play a key role in bolus formation, dynamic viscoelastic properties of human saliva have been studied^75^ and then measurements of primary normal force originating from the elastic nature were carried out for saliva collected before and after acid and mechanical stimulation.^76^ More recently, the pronounced elasticity of human saliva was also observed during elongational experiments on xanthan solutions.^77^ The breakup time of a 0.5% xanthan solution in the presence of human saliva was significantly increased when compared with the same xanthan solution in the presence of artificial saliva (alpha-amylase solution), showing that human saliva may have a positive impact on the elasticity of the food bolus during swallowing. Nevertheless, studies involving real human saliva are complex and difficult to generalize due to high variability of viscoelastic properties of saliva from individual to individual.

Despite the simplicity of the salivary solution used in this representation, the results obtained provide a first view of the impact of salivary alpha-amylase on both, shear and elongational properties of dysphagia-designed fluids containing different types of thickener, differentiating gum-based and starch-based fluids when considering their capacity to withstand enzymatic degradation, and therefore, to conserve their cohesiveness. Capillary break-up rheometry has been found to be a quick and sensitive method to characterize the structure changes in the presence of alpha-amylase, which could have a significant importance in aspects related to swallowing safety.

In addition to this, yield stress is another interesting fluid property that could play a role in understanding cohesiveness. Nakauma et al.^78^ found that the yield stress of XG solutions is much higher than that of LBG solutions at equivalent concentrations. The yield stress has been a matter of debate because the measurement of the viscosity at very low shear rate is difficult, and even at a very low shear rate the viscosity defined as the ratio of stress to shear rate was found to continue to increase with decreasing shear rate for xanthan, that is, shear thinning^79^.

**Fig. 3:**
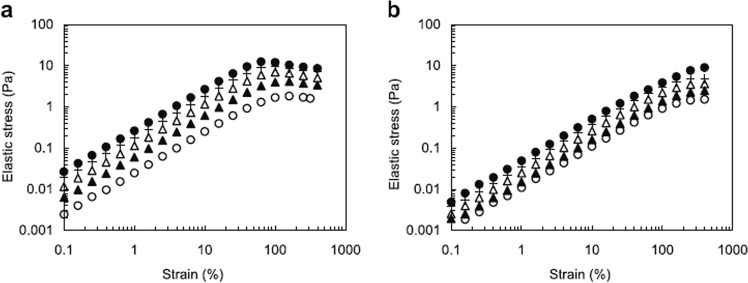
Elastic stress (storage modulus × strain) of xanthan gum and locust bean gum solutions at 20 °C and angular frequency = 6.28 rad/s.plotted as a function of strain. Concentrations of polysaccharide: 0.3, 0.45, 0.6, 0.75 and 0.9% for xanthan gum (**a**) and 0.5, 0.6, 0.7, 0.75, and 0.8% for locust bean gum (**b**), represented by open circles, closed triangles, open triangles, crosses, and closed circles in increasing order for each polysaccharide. The yield stress and strain were estimated from the maximum point in the curve^78^

Although it is not easy to be measured, the concept of yield stress is meaningful in food science and technology.^60,80–82^ Nakauma et al.^78^ using a method proposed by Walls et al.^83^ determined the yield stress of XG, that is, the yield stress is determined from the maximum in plot of the elastic stress, the product of the storage modulus and the strain, vs. strain (Fig. 3a). The yield stress of xanthan solution was found to increase with increasing concentration of xanthan, from 1.5 Pa for 0.3% XG to 10 Pa for 0.9% XG, while LBG solution did not show such a maximum in a similar plot (Fig. 3b) indicating that yield stress of LBG is negligible compared to XG.

From both, extensional behaviour and yield stress measurements, it is expected that xanthan solutions are much more cohesive than those of galactomannans, such as LBG. Cohesiveness seems to be attributed to the internal structure of the fluid which maintains its integrity during different types of deformation, and may be characterized by extensional deformation and force at break in a fluid extensional experiment. The yield stress seems to be, as well, highly correlated, but in order to clearly understand to which extent these properties can be interconnected, a quantitative examination is required in the near future.

## Acoustic analysis of swallowing of gum-based fluid models

Acoustic analysis of swallowing has been used extensively since it is non-invasive and more convenient technique than videofluorography (VF). The sound detected by a miniature accelerometer or microphone fixed near the vocal chords during swallowing is analysed. The identification of the event in the sound signal during swallowing has been done with simultaneous recording of videographic tapes of swallowing.^84,85^

Nakauma et al.^78^ compared the flow behaviour of XG solutions (0.3-0.9%) and LGB solutions (0.5–0.8%) in swallowing, by using acoustic analysis. Swallowing sound was recorded using a throat microphone, and the data obtained were processed by the wavelet transformation in order to obtain the frequency distribution.

**Fig. 4:**
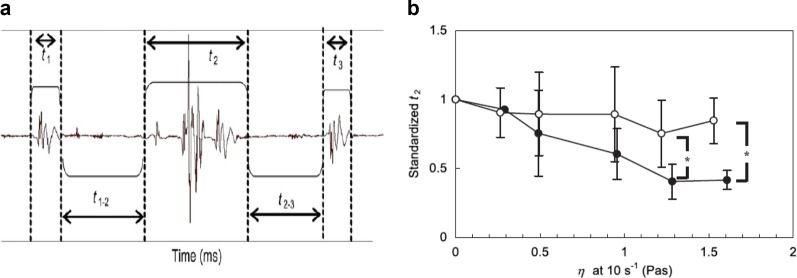
**a** Representative profile of the swallowing sound in the case of 15 mL water (20 °C). *t*_1_, the closure of the epiglottis; *t*_2_, the bolus flow; *t*_3_, the opening of the epiglottis in order of occurrence. *t*_1-2_, time interval between the termination of the epiglottis closure and the initiation of the flow of bolus; *t*_2-3_, time interval between the termination of the flow of bolus and the initiation of the epiglottis opening. **b** Duration *t*_2_ in swallowing polysaccharide solutions as a function of steady shear viscosity at 10/s. closed circle: xanthan gum; open circle: LBG. Serving volume of polysaccharide solutions was 15 mL. Each datum was standardized with that for control (water). Data with asterisk are significantly different between xanthan gum and LBG at *p* < 0.05^78^

A representative profile of the swallowing sound (Fig. 4a) was divided into three parts, which were assigned to the closure of the epiglottis (*t*_1_), the bolus flow (*t*_2_), and the opening of the epiglottis (*t*_3_) in the order of occurrence.^85^ To compare the swallowing behaviour of XG and LBG solutions, the standardized flow time is plotted as a function of the viscosity at 10/s in Fig. 4b. Here, the flow time is represented taking water as a reference, that is, each *t*_2_ is standardized by the time of water swallowing. Since a bolus tends to form a lumped mass with increasing concentration of XG, *t*_2_ for XG becomes shorter at higher concentration of XG. On the other hand, since LBG would not form structure irrespective of concentration, *t*_2_ for LBG at any range of concentration is similar to that for water. For xanthan solutions, the bolus flow time decreased with increasing the viscosity, that is, with increasing xanthan concentration while it was less concentration dependent for LBG solutions.

The decrease in the bolus flow time (*t*_2_) for xanthan solutions with increasing concentration, is counterintuitive because the flow velocity in the pharynx decreases with increasing concentration, according to an ultrasound Doppler analysis.^86^ The decreasing tendency of *t*_2_ with increasing concentration of xanthan was most remarkable for 15 mL serving than for 5 mL serving.^78^

**Fig. 5:**
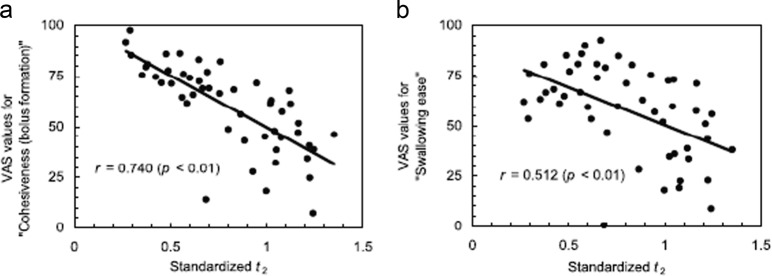
Correlation between the duration *t*_2_ and the score of each sensory attribute in swallowing polysaccharide solutions. Serving volume of polysaccharide solutions was 15 mL. **a** Sensory cohesiveness (bolus formation); **b** Sensory swallowing ease. The *t*_2_ was standardized with the corresponding data for water. Sensory evaluation was represented by a visual analogue scale (VAS)^78^

The reason for the flow time *t*_2_ decrease with increasing viscosity (or concentration) of xanthan is attributed to the increased internal structure or cohesiveness for xanthan solutions. While the control (water) showed splashing and a broad distribution of each bolus fragment both in an ultrasonic pulsed-Doppler method^86^ and in finite element analysis^87^ of swallowing, XG showed a cohesive bolus with a narrower velocity profile. The significant difference between the flow times for 15 mL XG and LBG solutions detected by the analysis of the swallowing sound shown in Fig. 4b cannot unfortunately be compared directly with the results of Kumagai et al.^86^ who used a serving mass 6 g, when the effect of the serving volume is taken into account. When the serving volume increased from 5 to 15 mL, for water, the flow time *t*_2_ increased from 56.0 to 85.9 s while for 0.75% xanthan solutions *t*_2_ decreased from 49.8 to 34.8 s. The decrease in bolus flow time with increasing viscosity of xanthan is counterintuitive, as mentioned above. It should be noted that the velocity of the bolus reported by Kumagai et al.^86^ is the value for a part of the bolus irradiated by ultra sound, while in the measurement of Nakauma et al.^78^ the flow time *t*_2_ is the elapsed time from the arrival of the bolus head until the leaving of the bolus tail. Therefore, the time required is shorter for cohesive bolus such as XG and longer for less cohesive LBG.^86^ showed narrower distribution for a more viscous bolus than for a less viscous one, suggesting that more viscous samples are more cohesive. This is consistent with the observation of Nakauma et al.^78^ that the time *t*_2_ is shorter with increasing concentration for xanthan because cohesiveness increases with increasing concentration (Fig. 5a), which is also in good agreement with Turcanu.^64^

The pharyngeal phase transfer time (also represented by *t*_2_) becomes shorter with increasing XG concentration. This suggests two possibilities. The first one is that the flow velocity of xanthan gum in solutions increases with concentration. This is not the case because concentration increase brings in the viscosity increase, and increased viscosity decreases the flow velocity of solutions when the same amplitude of stress is applied, as already shown by Kumagai et al.^86^

When the cohesiveness and the ease of swallow, evaluated by sensory evaluation, are plotted as a function of the flow time, both, increased with shortening of the flow time. The cohesiveness is taken as the formation of the bolus, and the easiness of swallow coincides with the easiness of the bolus formation (Fig. 5b). This cohesiveness is related to the “structured fluid” or “weak gel” nature of xanthan gum, which promotes the formation of one internally structured bolus that could be swallowed integrally, at once. It should be noted here that the concept of cohesiveness determined in a conventional TPA analysis cannot be applied to solutions of thickening agents, such as xanthan, as already mentioned before.

To compare the acoustic analysis with the sensory evaluation of adhesiveness, cohesiveness and the ease of swallow, the acoustic balance, *r*_2_, of the swallowing sound, defined as the ratio of sound pressure (in dB) at a high frequency (3.0–4.5 kHz) range to that at a low-frequency (0.3–0.9 kHz) range was found to be a good index to understand the relation.^78^ Since the acoustic balance, *r*_2_, increased with increasing concentration of xanthan, both the cohesiveness and the ease of swallow increased with increasing *r*_2_.

The cohesiveness of bolus plotted as a function of flowing time in Fig. 5a and as a function of acoustic balance (shown in Nakauma et al.^78^) could be related with the elongational breakup time, which is the filament ability to withstand the capillary breakup in the uniaxial elongation, as shown in Fig. 1. A longer breakup time and higher filament ability could be related to a higher cohesiveness.

Rheological characteristics required for thickeners in dysphagic treatment seem to be a so-called weak gels or structured fluids which have a high cohesiveness. Therefore, it would be necessary to study in more details the relation among cohesiveness, extensional viscosity and yield stress.

## VF observation of swallowing liquids with different degrees of shear thinning

The X-rays videofluorography (VF) is thought to be a golden standard in dysphagia treatment because it can visualize the swallowing process of the bolus containing contrast medium, compounds containing heavy atoms such as barium or iodide. However, these contrast media affect the rheological properties, particularly when polyelectrolytes, like carrageenan and gellan gum, are used. Dependence upon the equipment, personnel, and scheduling of the radiology suite tends to reduce the efficiency of diagnosis and management. It is also inaccessible to patients who are physically unable to move to the radiology suite. Reliance on X-irradiation is of concern for patients who need frequent reassessments of swallowing function, as after cerebro-vascular accident or head and neck cancer surgery. In addition, the procedure is expensive, as was pointed out by Bastian.^88^

**Fig. 6:**
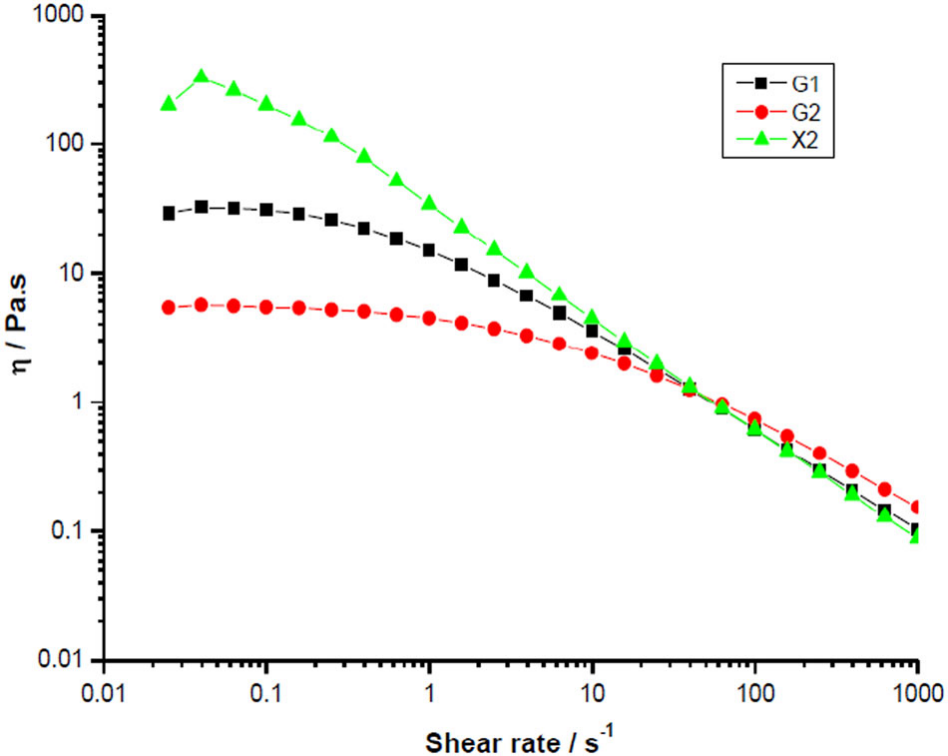
Shear rate dependence of the viscosity of xanthan X2 and guar (G1 and G2) solutions containing iopamidol. The measurement temperature: 36.5 °C. The shear rate was changed stepwise up to 1000/s for 10 min^89^

VF observations of swallowing of three different liquids by 32 patients were carried out by the direction of Michiwaki in Japanese Red Cross Musashino Hospital. Three polysaccharide solutions which show the same steady shear viscosity at 50/s, but different shear thinning behaviours as shown in Fig. 6.^89^ The shear rate 50/s was chosen because it was widely used since American National Dysphagia Diet (NDD) proposed it in 2002^22^ although there have been many debates on this value. More information regarding this topic is given in the section: Shear rate in oropharyngeal region.

Three-sample solutions were used. Sample 1 (G1), 2.0 wt% GG solution stirred at room temperature for 10 h, then heated at 75 °C for 1 h (*M*_w_ = 1.4 × 10^6^, *M*_w_/*M*_n_ = 1.2). Sample 2 (G2), 2.6 wt% GG solution stirred at room temperature for 10 h, then heated at 75 °C for 1 h and sterilized at 121 °C for 20 min (*M*_w_ = 6.3 × 10^5^, *M*_w_/*M*_n_ = 2.1). Sample 3 (X2), 4.1 wt% xanthan solution stirred at room temperature for 10 h, then heated at 75 °C for 1 h and sterilized at 121 °C for 20 min (*M*_w_ = 3.4 × 10^6^, *M*_w_/*M*_n_ = 1.6). The contrast medium chosen was Iopamidol (Ioverin 300, Taiyo Pharmaceutical Industry, Co. Ltd, Nagoya) which was affirmed to be non-toxic for lungs. The xanthan based solution shows a shear thinning behaviour in the wide range of shear rate as shown in Fig. 6, that is, the steady shear viscosity decreases conspicuously with increasing shear rate, as already well known.^67,90^ The apparent shear thickening observed for xanthan solutions at lower shear rates in Fig. 6 has been also seen by some workers,^91,92^ and it may be attributed to some structural formation during the quiescent storage, which may take a longer time and the system is not in equilibrium because this shear thickening was not found when the shear rate was lowered. Recently, Wagner et al.^93^ reexamined this problem, and proposed two criteria to ensure equilibration during steady state flow rheological measurements: a substantial increase in the measurement time allotted for each point such that the total material strain accumulated in the sample is allowed to reach shear strain ≤5 and/or a stricter convergence criterion of 10 consecutive readings within a tolerance of 1%.

Solutions of GG show also shear thinning behaviour. It is known that the shear rate at which the shear thinning starts depends on the dispersity *M*_w_/*M*_n_ (previously called polydispersity) of the polymer, and that the polymer with narrow molar mass distribution (MMD) moves from a well-defined Newtonian region to power-law behaviour over a narrower range of shear rate than the polymer with broader MMD.^67^ As written above, the dispersity of G1 and G2 were 1.2 and 2.1, respectively, and the decrease of the viscosity with increasing shear rate was more gradual in G2 than G1, which is consistent with the general tendency mentioned above. Note that the order of the magnitude of the viscosity at higher shear rate and that at lower shear rate is opposite: *η*_LS_ (X2) > *η*_LS_ (G1) > *η*_LS_ (G2) at lower shear rate, but *η*_HS_ (X2) < *η*_HS_ (G1) < *η*_HS_ (G2) at higher shear rate.

**Fig. 7:**
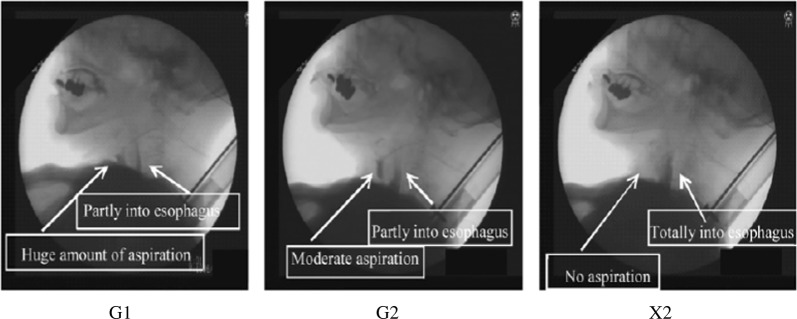
Videofluorography of a patient who showed aspiration for two sample solutions G1 and G2, and without aspiration for X2^89^. The videofluorographic observation described in the present paper was approved by Ethic Committee of Musashino Red Cross Hospital in accordance with the Declaration of Helsinki

Figure 7 shows a typical example of VF observation of a patient during the deglutition. For this case, no aspiration occurred in X2, which has the highest viscosity at lower shear rates. For GG solutions, G2 solutions showed moderate aspiration while G1 showed a huge aspiration. The magnitude of the viscosity was as follows: *η*_HS_ (G2) > *η*_HS_ (G1) at higher shear rates although *η*_LS_ (G2) < *η*_LS_ (G1) at lower shear rates. However, the hypothesis that a liquid with higher viscosity at higher shear rate is effective to prevent the aspiration in the case of GG solutions is contradictory to the above judgement for xanthan solution which showed the highest viscosity at lower shear rate but the lowest viscosity at higher shear rate. Although the difference in the viscosity at lower shear rate is very distinct, that at higher shear rate was not so distinct. When we summarize all 32 observations, the number of aspirations was 4 for X2, 5 for G1 and 7 for G2. Thus, there is a general tendency that the viscosity at lower shear rate seems to be more important rather than that at 50/s or higher shear rate. The probability of the aspiration seems to decrease with increasing the viscosity at lower shear rate. Since the number of observation is not sufficiently high, this supposition should be confirmed with larger number of data in the future. It should be mentioned here that when the concentration of GG was higher to increase the viscosity at lower shear rates, panellists found the difficulty to swallow which is in line with Vickers et al.^94^ as mentioned below.

Extensional viscosity and the breakup time for the filament of polymer solutions is shown to increase with increasing molar mass of the polymer in the capillary breakup experiment.^95^ The experimental finding that the probability of aspiration was lowest for xanthan was tentatively attributed to the highest viscosity at lower shear rate, but it should be also attributed to the highest cohesiveness as described above. The difference in the aspiration probability between lower molar mass guar gum G2 and higher molar mass G1 might be also related to the lower cohesiveness of G2 than G1 because the cohesiveness is characterized by the extensional behaviour as mentioned earlier.

As for the effect of the medical history on the occurrence of the aspiration, it is the problem in the realm of medicine and it is under the investigation by Michiwaki. This study showed the possibility to find what kind of fluid induces the aspiration and to reduce the risk of aspiration in dysphagic patients by controlling the food rheology. But many other works should be done in the future. VF from one direction gives only a two-dimensional information. Not only the rheology (texture) but also the flavour should be combined with texture to make a solution for judging the risk of aspiration and for real food design.^3^

Vickers et al.^94^ notice the importance of sensory evaluation because the swallowing behaviour is influenced by the thickness, stickiness, adhesiveness, and mouth coating. Using nectar like beverages with the viscosity 300 mPas at 30/s and thin honey like beverages with the viscosity 1500 mPas at 30/s, they performed the sensory evaluation and compared with steady shear viscosity measurements. They suggested that the fluids with relatively high shear thinning are more suitable for successful swallowing, which is closer to the conclusion of Nishinari et al.^89^ They stated that more shear thinning liquids were generally perceived as less thick, with less adhesive properties (stickiness, adhesiveness, mouth coating, and number of swallows) and greater slipperiness. This is consistent with the observation of Nakauma et al.^78^ and the panelists’ feeling that a xanthan solution was easier than a GG solution when the viscosity was made the same at 50/s. Vickers et al.^94^ defined the mouth coating as the degree to which the beverage coats the inside of the mouth after swallowing, and quantified it by measuring the residual riboflavin. One gram per litre of riboflavin was incorporated into the base liquid of all the thickened beverages during preparation, and the amount of riboflavin left in the mouth plotted against the sensory mouth coating scores showed a linear relationship. They concluded that a desirable thickener for many dysphagic patients would be one that allowed for a safe swallow (the fluid would have rheological properties that reduced airway penetration) yet be pleasant to drink (minimal perceived thickness, minimal mouth coating, and minimal off flavours). The ease of swallow may be closely related or very similar to the pleasantness to swallow, which means less adhesive and less mouth coating and also related with high cohesiveness.

## Shear rate in oropharyngeal region

It is important to know the shear rate in the oral and pharyngeal phase to design liquids safe to swallow and pleasant to drink although 50/s has been widely used, including the tentative proposal of the International Dysphagia Diet Standardization Initiative (IDDSI).

Shama and Sherman^96^ proposed a universal shear stress-shear rate curves based on instrumental measurements of viscosity and sensory evaluations of thickness of various liquids. They found that thin liquids of which the viscosity is lower than 0.1 Pas are swallowed at a low stress of 10 Pa, while thick liquids of which the viscosity is higher than 1 Pas are swallowed at a low shear rate of 10/s by increasing shear stress with increasing viscosity. Then, Kokini et al.^97^ calculated the shear stress when viscous liquids are subjected between the tongue and the hard palate, and compared the calculated results with the universal curve of Shama and Sherman.

Houska et al.^98^ re-examined this problem using five different methods of evaluation of thickness for model and real Newtonian liquid foods: (a) effort exerted in swallowing the sample, (b) force necessary to compress the sample between tongue and palate, (c) effort to slurp the sample from a soup spoon into the mouth, (d) manner in which the fluid poured back into the beaker from a soup spoon, (e) effort necessary to mix with a spoon 50 mL of the fluid in a 150-mL beaker. They found that the highest shear rates for viscosity perception by compression of samples between tongue and palate, and the lowest for pouring the fluid foods from a teaspoon. General tendency was similar to the universal curve of Shama and Sherman;^96^ however, they found that the shear rate tended to farther slower rate while Shama and Sherman predicted that the shear rate converged to 10/s with increasing viscosity of the liquids.

Recently, Sonomura et al.^87^ modelled the movements of the tongue and pharynx to simulate the swallowing flow. The interaction of the flow of the bolus and the movements of the pharynx were analysed using a finite element method. Solutions of xanthan and guar used in Nishinari et al.^89^ were used as model boli, which were well approximated by power law models. Two kinds of abnormal swallowing were analysed numerically: (1) when the disorder was caused by dysfunction in the lift up of the throat and retroflexion of the epiglottis, the water bolus was scattered over the epiglottis and was partly aspirated. (2) When the disorder was caused by early termination of swallowing movements, a part of the bolus remained around the epiglottis, and the return of the epiglottis induced part of the bolus to drop into the larynx. These results suggested that the pattern of the aspiration depended on the swallowing movements, and the viscosity and volume of the bolus.

In the study of the saltiness intensity and the viscosity of the polysaccharide (xanthan or GG) solutions with salt, the higher correlation was found between saltiness intensity and the viscosity measured at shear rates higher than 100/s was observed.^99^ It was then concluded that the shear rate at which the thickness was perceived in the mouth flow is higher than 100/s. Kumagai et al.^86^ found that the maximum velocity at pharynx measured by Doppler method decreased with increasing concentrations of all thickeners, and the correlation was found highest when the viscosity at the shear rate of 10/s was used in the examination of the relation between the velocity and the viscosity.

For the study of swallowing safety, it is necessary to know the shear rate in the pharyngeal region rather than in the mouth. Brito-de la Fuente et al.^100^ reported swallowing shear rates from bolus transit velocities and got 931.7/s for bolus head and 262/s for bolus tail in pharyngeal phase and much lower value for oesophageal phase using previous data on the geometry of oral anatomy. Salinas-Vazquez et al.^101^ studied the peristaltic flow through the pharynx from the glossopalatal junction to the upper oesophageal sphincter numerically to find the causes leading to abnormal swallowing. They observed that the bolus head travels faster than the bolus tails, which indicates that the bolus is also subjected to extensional stresses, which agreed well with previous findings of Bardan et al.,^102^ who reported 37.6 ± 8.1 and 10.3 ± 3.0 cm/s for the bolus head and tails in pharyngeal phase, respectively. Zhu et al.^103^ found that the bolus velocity was faster in meso-pharynx than in hypopharynx, and proposed characteristic shear rate 120/s in meso-pharynx and 990/s in hypo-pharynx irrespective of the viscosity examined (0.01–1 Pas).

More recently, Ong et al.^104^ suggested that a single oral shear rate could not be used to predict perceived viscosity and hypothesized that panellists might need to use different methods to evaluate oral perceived viscosity of different viscosity levels. This seems to be in line with a mouth model of two compartments, consisting of a well-mixed region in the bulk of the mouth and a non-mixed diffusion-controlled region close to the tongue, proposed by Le Reverand et al.^105^ It is unfortunate that the labels of their figures are wrong, and the errata will be published soon.

Although the shear rate in the oropharynx is still not completely well defined, its importance in the realistic evaluation of swallowing and aspiration is unquestionable.

## Conclusion

Cohesiveness, in addition to the viscosity, is shown to be an important index in the evaluation of thickened fluids for dysphagia treatment. In gum-based models, cohesiveness was shown to be highly correlated with high extensibility and finite yield stress, and shown to be related to the presence of the elastic behaviour. This is consistent with the experimental findings that xanthan has a higher extensibility and a finite yield stress compared to locust bean gum which has a very weak extensibility and no yield stress. In addition to extensibility, cohesiveness is thought to be partially correlated with a high yield stress, but this should further be examined quantitatively. According to these findings, it can be hypothesized that highly cohesive fluids are less fragmented into smaller particles in the oropharyngeal and oesophageal region and are safer than fluids with lower cohesiveness.

Nevertheless, the swallowing process is still a very complex and, additionally to the bolus structure and rheology, can be influenced by many other physiological, psychological and chemical factors. It is well known that chemical senses also affect the swallowing behaviour,^3,106,107^ but the interaction between cohesiveness and chemical senses, such as taste and aroma, is still not well understood and should further be studied. Since the rheological properties such as extensional behaviour and yield stress depend on molar mass, detailed structure and concentration of xanthan and galactomannans, more detailed studies using solutions of these well-defined polysaccharides are required. It is also necessary to study the droplet size distribution just after the ejection of the fluid from a nozzle to see the correlation with the fluid extension experiment and the yield stress and to understand better the cohesiveness. Since fluid gels, emulsion gels, aerated gels might seem also to be promising tools to modify the texture of foods for dysphagia, the cohesiveness for these materials should be also studied. Additionally, although other methods, such as back extrusion or vane geometry have already been used, as far as the authors are aware, the elongational rheology of non-homogenous or unstable fluid suspensions with partially soluble and swollen particles have not been yet addressed. This is also another important problem related to a better understanding of the bolus rheology.

## Data Availability

The authors declare that the data supporting the findings of this study are available within the paper.
